# Maximum Data Collection Rate Routing Protocol Based on Topology Control for Rechargeable Wireless Sensor Networks

**DOI:** 10.3390/s16081201

**Published:** 2016-07-30

**Authors:** Haifeng Lin, Di Bai, Demin Gao, Yunfei Liu

**Affiliations:** 1 College of Information Science and Technology, Nanjing Forestry University, Nanjing 210037, China; haifeng.lin@njfu.edu.cn (H.L.); gdmnj@163.com (D.G.); lyf@njfu.edu.cn (Y.L.); 2 College of Engineering, Nanjing Agricultural University, Nanjing 210009, China; 3 School of Computer Science and Engineering, Southeast University, Nanjing 211189, China

**Keywords:** wireless sensor networks, maximum data collection Rate protocol, topology control, data aggregation, routing protocol, rechargeable-WSNs

## Abstract

In Rechargeable Wireless Sensor Networks (R-WSNs), in order to achieve the maximum data collection rate it is critical that sensors operate in very low duty cycles because of the sporadic availability of energy. A sensor has to stay in a dormant state in most of the time in order to recharge the battery and use the energy prudently. In addition, a sensor cannot always conserve energy if a network is able to harvest excessive energy from the environment due to its limited storage capacity. Therefore, energy exploitation and energy saving have to be traded off depending on distinct application scenarios. Since higher data collection rate or maximum data collection rate is the ultimate objective for sensor deployment, surplus energy of a node can be utilized for strengthening packet delivery efficiency and improving the data generating rate in R-WSNs. In this work, we propose an algorithm based on data aggregation to compute an upper data generation rate by maximizing it as an optimization problem for a network, which is formulated as a linear programming problem. Subsequently, a dual problem by introducing Lagrange multipliers is constructed, and subgradient algorithms are used to solve it in a distributed manner. At the same time, a topology controlling scheme is adopted for improving the network’s performance. Through extensive simulation and experiments, we demonstrate that our algorithm is efficient at maximizing the data collection rate in rechargeable wireless sensor networks.

## 1. Introduction

Recent years have witnessed the emergence of a significant promising technology, Wireless Sensor Networks (WSNs), containing numerous inexpensive sensor nodes randomly scattered over the area of interest to collect information on entities of interest. WSNs have been used for wide-ranging applications, such as in the military [1], environmental [2], health [3] and smart home fields [4]. In WSNs, sensor nodes are generally equipped with limited energy battery and limited power storage with low power, small size and low cost [5]. Sensor nodes are usually deployed in remote or dangerous areas. They can sense ambient conditions and generate data to reflect the real-world scenario [6]. Sensors can communicate with each other over a short distance and relay information on a wireless transmission device. Through multiple hop transmission, this information can be transmitted to a remote terminal and processed by other devices.

In WSNs, since researchers have not made breakthrough progress in battery technology, a fundamental problem is the limited lifetime of sensor nodes due to limited and fixed energy supplement [7,8]. Note, it is generally accepted that the usefulness of a wireless sensor expires when its battery runs out. A significant amount of work has been carried out across the protocol stack to prolong the sensor’s lifetime by saving energy [9,10], including duty-cycling strategies [11,12] and Medium Access Control (MAC) protocols [13,14]. However, it is inevitable that the energy will be exhausted and sensors have to be discarded because it is generally impractical to render servicing for sensors. Meanwhile, it is inevitable that the accumulation of discarded batteries will contaminate the environment.

**Figure 1:**
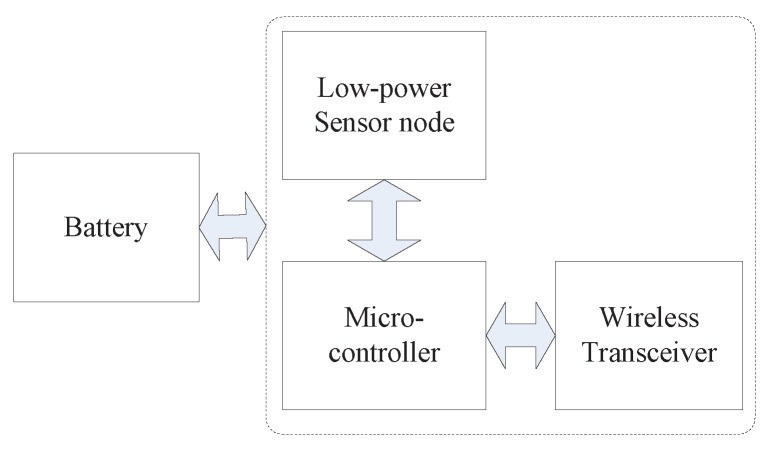
Components of a battery-powered sensor node.

Currently, Energy Harvesting or Rechargeable Wireless Sensor Networks (EH-WSNs or R-WSNs) have attracted more and more attention. They benefit from extending the lifetime of sensor nodes by equipping them with rechargeable technologies [15,16], which convert sources, such as body heat [17], foot strike [18], finger strokes [19], and solar [20] into electricity. A sensor can operate perpetually by using supercapacitors (with virtually unlimited recharge cycles) to store the harvested energy [21]. Note, a harvesting node is said to achieve energy-neutral operation if the energy used is always to a lesser degree than the energy harvested [22]. The energy rechargeable device and energy storage device as the energy supplement components of R-WSNs replace the common accumulator battery that is used in traditional WSNs (T-WSNs), as is shown in Figure 1, in T-WSNs, the the system architecture contains four components: battery, low-power sensor node, micro-controller and wireless transceiver device.

**Figure 2:**
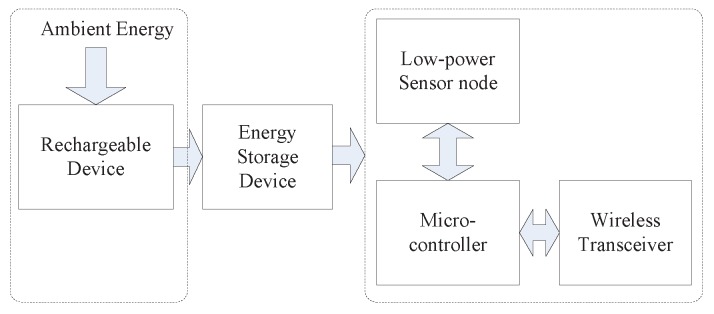
Components of a energy-powered sensor node.

In the R-WSNs, the system architecture contains five parts: rechargeable system, battery, low-power sensor node, Micro-controller and Wireless Transceiver device. The rechargeable system is utilized to extract the energy from ambient conditions, which is the key difference between the new WSNs and T-WSNs. The system architecture of a rechargeable sensor node is illustrated in Figure 2. Although their lifetime is less of an issue, a sensor node has to confront two issues. In R-WSNs, the energy available is usually very dynamic, which is influenced by the environment and can vary significantly over time. First and foremost, owing to the restriction of development of conversion technology, the sensor has to stay in a dormant state most of the time in order to recharge the battery and use the energy prudently. Moreover, due to the limited energy storage capacity, a node cannot always be beneficial at conserving energy when a network can harvest excessive energy from the environment [23,24]. Since more energy can be extracted from the ambient environment in R-WSNs, the harvested energy should be consumed as soon as possible [25,26]. Therefore, the surplus energy of a node can be utilized for strengthening packet delivery efficiency and improving network data collection rate.

Data aggregation can reduce data traffic significantly by removing data redundancy [27]. Since different sensor nodes partially monitor the same spatial region, sensing information is generally correlated [28]. To saving power, data should already be processed as it flows from the information source to the sink [29], which will decrease the accuracy of information around the monitoring area due to some relative packets being removed [30]. Therefore, we should let the sink collect more data only if its energy can support the action [31]. However, more packets indicate that more energy will be depleted for generating and forwarding packets. Hence, data aggregation technology can be utilized to deal with packets compression prudently [32]. More packets collected at the sink means more accuracy of the information and more energy needed. However, data aggregation causes less packet transmission, energy depletion and accuracy. Energy expenditure and data accuracy will be traded off before utilizing data aggregation for multiple-hop transmission in WSNs and R-WSNs.

Topology control is used for energy conservation of nodes or energy utilization reasonably, by shifting the roles of nodes from active to sleeping state and active state [33], which is significantly different to traditional WSNs. In T-WSNs, the topology control technique also is used for handling the limited energy of sensor [34], for example, if a sensor node has not been assigned new tasks, it will shut down the wireless transceiver device and stay in an idle state to save energy [35]. When a task coming, the sensor node wakes up itself and shifts into an active state for dealing with the issues [36]. However, it is inevitable to cause serious data transmission latency due to frequent shifts into the idle state in T-WSNs. In contrast, in R-WSNs, a sensor node does not shift into sleeping state when no task is assigned, but only if it has enough energy to support its operation. Therefore, in R-WSNs, topology control is utilized flexibly compared to the use of energy in T-WSNs.

In summary, we propose a maximum data collection rate routing protocol based on data aggregation for packets communications in R-WSNs. To the best of our knowledge, this is the first generic work that studies the use of distribute algorithm for maximizing data collection rate based on data aggregation technology in R-WSNs. In the work, we compute an upper data generation rate by maximizing it as an optimization problem and energy definition as constraint condition for a network, which is formulated as a linear programming problem. Subsequently, a dual problem by introducing Lagrange multipliers is constructed, and subgradient algorithms are used to solve it in a distributed manner. At the same time, a topology controlling scheme is adopted for improving network performance. The remainder of this paper is organized as follows. In Section 2 we present a number of existing routing protocol based on data aggregation, while in Section 3 specify the network model and assumption. In Section 4 we present our method and design. Section 5 contains experimental results. Conclusions are presented in Section 6.

## 2. Related Work

The data collection rate is affected significantly by the energy extracting rate from the environment. Therefore, a large number of researchers pay the utmost attention to the characteristics of R-WSNs [37,38], especially regarding the features of energy supplement. At present, solar is the main energy source considered firstly for providing clean power in R-WSNs and a solar panel is generally installed on the outside to recharge an energy storage device for a sensor node [39]. On the one hand, considering the cost factors and economic feasibility, the size of the solar panel always is small, which results in limited energy being gained [40]. On the another hand, considering the weather factor and the different angles of the solar panel, each sensor node will obtain distinct power. Thus, it is not realistic the achieve data transmission synchronously in R-WSNs. For maximizing data collection rate, we analyze the techniques provided in traditional WSNs.

Numerous works have been done focusing on maximizing data generation rate by saving energy in WSNs; e.g., [41,42,43]. A degree-constrained routing tree is introduced by minimizing hop counts with transmission power control to enhance the data collection rate, which can avoid the high-degree bottleneck effectively [44]. In [45], the authors present the optimal convergecast scheduling algorithms by the maximum degree in the routing tree for which information is collected fast. Chen et al. [46] provide an iterative linear programming solution for finding the maxmin optimal rate assignment in a low-rate sensor network. Su et al. [47] formulate the optimal rate allocation with data aggregation as a network utility maximization problem and apply duality theory to decompose it into a rate control subproblem, other examples can be found in [48,49], and references therein. Despite the maximum data generation rate achieved in these algorithms, they encounter critical tradeoffs between data flow and lifetime due to energy constraint. In R-WSNs, the lifetime is less of an issue, which can be maximized by operating in an energy neutral mode for nodes.

Regarding the characteristics of R-WSNs, several protocols discuss several aspects of power management or MAC schemes to improve energy efficiency and maximum data collection rate [50,51]. A centralized algorithm with line programming is proposed to compute the lexicographically maximum data collection rate and routing paths for each node [52]. Sadlapur et al. [53] provide a distribute algorithm for jointly determining the routing structure and amount of flows on each link with flow adjustment to achieve an optimal data collection rate. Peng et al. [54] propose real time adaptive energy management policies based on observed information for optimal throughput. Prabhakar et al. [55] propose four throughput enhancement schemes from a simple naive scheme with low complexity to a probabilistic probing scheme incorporating advanced methods to appropriately use the harvesting energy. However, the ideal energy replenished precondition is used and data aggregation has not been considered in these protocols.

Data aggregation is adopted widely in WSNs for improving data collection rate by removing redundant information [56]. The main idea behind data aggregation or data fusion is that, rather than sending individual data items from sensors to sinks, multiple data items are aggregated as they are forwarded by the sensor network [57]. In [44], the authors introduce two strategies to maximize the data collection rate: an aggregation tree is contracted to minimize the number of time slots needed and combine the scheduling with transmission power control to reduce the effects of interference. Demin et al. [58] introduce an algorithm for maximum data collection rate in rechargeable wireless sensor networks with multiple sinks. In [59], the authors present a fuzzy-based data fusion approach for increasing the QoS whilst improving the data collection rate and reducing the energy consumption of the sensor network.

The topology control mechanism is utilized widely for reducing energy consumption to prolong the network lifetime by predicting the topology control messages in WSNs [60]. For coping with the data collection with delay-constraint, a load-aware power-increased topology control algorithm is presented to heuristically solve the problem, which reaches the O(1)-approximation ratio for the linear networks [61]. A new topology control mechanism, called hierarchical neighbor graphs, is proposed to build a structure for data collection depending on a single parameter, which requires only local knowledge at each node to be formed and repaired [62]. In [63], the authors propose approaches to maximize the topological network lifetime of the WSNs by considering a two-tiered Wireless Sensor Network deployed around strategic locations and base-stations. These techniques with topology controlling schemes are utilized for saving the energy and prolonging the network lifetime. Very few papers in the literature focus on the maximum data collection rate with data aggregation and topology control simultaneously in R-WSNs.

In contrast to earlier works, which either focus on static battery-powered network or maximum data generation rate without considering data fusion and topology control, in this work, we present a maximum data collection rate based on data aggregation and topology control for R-WSNs. In summary, on observing the lack of data aggregation and topology control techniques for improving data flow in existing routing protocols, we introduce the first generic routing protocol algorithm with the data aggregation and topology scheme in R-WSNs. To the best of our knowledge, there is no prior work studying the problem of maximum data generation rate routing protocol with data aggregation and topology control for R-WSNs.

## 3. System Model

### 3.1. Network Model

Consider a static rechargeable wireless sensor network modeled as an undirected graph G=(V, A), where *V* is the set of *n* rechargeable sensor nodes and sink nodes within the wireless sensor network. *A* is the set of links, A={A|(i, j)∈A, i, j∈V}. *G* consists of a finite nonempty vertex set *V* and edge set *A* of ordered pairs of distinct vertices of *V*. Two nodes *i* and *j* are connected by a link if they can transmit a packet to each other with a transmission power less than the maximum transmission power at each node, where a sensor node generally obtains a maximum transmission range depending on its maximum transmission power and neighbors only within the transmission range can receive the packets from the node. The maximum transmission power is affected by the hardware of wireless radio transmitting device. Considering the condition of energy supplement, at present, the maximum transmission range is set to be less than 200 m in the real scenario.

In the networks, all links are assumed to be bi-directional. This assumption is not necessary for the convergence of the distributed algorithms; however, it can make the presentation clearer. A graph is simple if it has no loops and no two of its links join the same pair of vertices. An acyclic graph is one that contains no cycles. The set of nodes are connected to node *i* by links denoted as $S_{i}$, which indicates that all sensor nodes in the set $S_{i}$ can communicate with sensor *i* directly without forwarding other sensor nodes. We assume that the network graph is connected, i.e., there always exists a path between any pair of nodes *i* and *j* in *V*. Isolated nodes are not considered in the work, even though they exist. The data generation rate and the remain energy of node *i* are expressed as $g_{i}$, $e_{i}$, respectively.

### 3.2. Energy Expenditure Model

The power consumption of a sensor node consists of five parts: sensing and generating data, idling, listening, receiving, and transmitting. The the power for generating one bit of data, from an idle state consumed by a node, is denoted by $e_{g}$ and $e_{idle}$, respectively. They are assumed to be the same for all nodes and independent of traffic. $e_{l}$ denotes the power expenditure for a sensor listening channel at each time point before sending a packet. For power consumption in receiving and transmitting, the first order radio model is adopted in [64]. Specifically, a node needs $\varepsilon_{elec}$ = 50 nJ for running the circuitry and $\varepsilon_{amp}$ = 100 pJ/bit/m${}^{2}$ for the transmitting amplifier. Therefore, the power consumption for receiving one bit of data is given by $e_{r}=\varepsilon_{elec}$. The power consumption for transmitting one bit of data to a neighbor node *j* is given by $e_{t}=\varepsilon_{elec}+\varepsilon_{amp}\times d_{i,j}^{n}$, where *n* is the path loss exponent, which typically ranges between 2 and 4 for free-space and short-to-medium-range radio communication. $d_{i,j}$ denotes the Euclidean distance of the node *i* and *j*. If the coordinate of node *i* and *j* are $\left(x_{i},y_{i}\right)$ and $\left(x_{j},y_{j}\right)$, respectively, the $d_{i,j}$ can be formulated as:

(1) $$d_{i,j}=\sqrt{{\left(x_{j}-x_{i}\right)}^{2}+{\left(y_{j}-y_{i}\right)}^{2}}$$

We assume the the data traffic from node *i* to *j* per unit time is $f_{i,j}$. The per unit time can be set to one second or one minute or one times data transmission in the work, which is decided by the requirement of experiment. The different units selected will not affect the performance and evaluation of the system. The energy consumption for node *i* in receiving and transmitting data flow $f_{i,j}$ are $e_{t}\left(i,j\right)$ and $e_{r}\left(i,j\right)$, respectively, which can be expressed as:

(2) $$e_{t}\left(i,j\right)=e_{t}\times \sum_{i,j\in V,j\in S_{i}}f_{i,j}$$

(3) $$e_{r}\left(i,j\right)=e_{r}\times \sum_{i,j\in V,j\in S_{i}}f_{i,j}$$

Let $w_{i}$ denote the total fraction of power consumption for node *i* per unit time. Considering the energy deplete for five parts, $w_{i}$ can be formulated as:

(4) $$w_{i}=e_{g}\times g_{i}+e_{idle}+e_{l}+e_{t}\left(i,j\right)+e_{r}\left(i,j\right)$$

### 3.3. Energy Replenish Model

**Figure 3:**
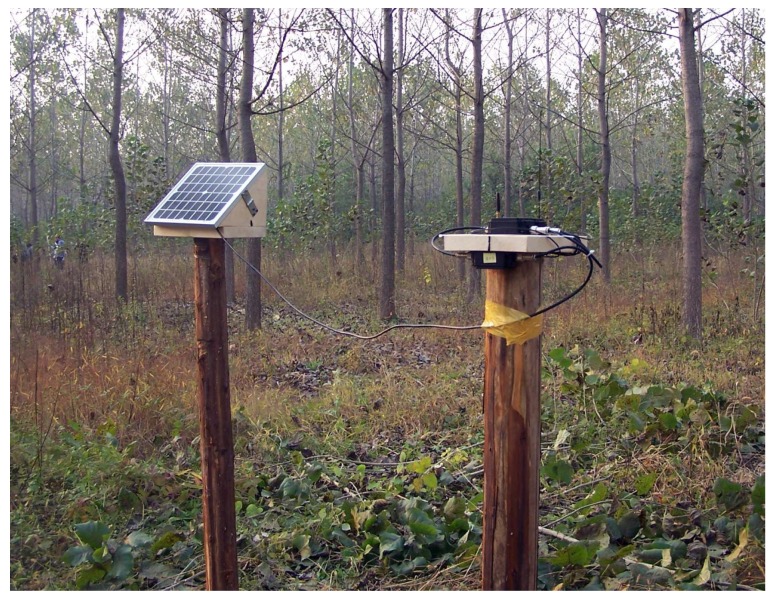
A sensor with solar panel is deployed in outside.

In R-WSNs, the energy extracting from ambient conditions varies with environment and weather conditions. Even though an energy source cannot be controlled to yield energy at the desired times, its behavior can be modeled to predict the expected availability at a given time. For a sensor power relying on solar power, as is shown in Figure 3, its energy cannot be controlled. However, models for its dependence on diurnal and seasonal cycles are known and can be used to predict availability. For a region, its weather parameter is usually similar to the history data of the same period. Suppose the power output from the energy source is $P_{i}\left(t\right)$ at time *t*.

In many instances, the energy generation profile may be very different from the consumption profile. To help support this scenario, consider a device that has a mechanism to store any energy that is harvested. The stored energy may be used at any time later. The energy buffer is defined to be a device that can store limited energy, has an inefficient storage in charging, and does not leak any energy over time. In this case, the energy harvesting from ambient for all non negative values of *T* can be calculated as:

(5) $$E_{i}=\int_{0}^{T}P_{i}\left(t\right)d\left(t\right)+B_{i},\forall T\in \left[0,\infty \right)$$

where, $B_{i}$ is the initial energy stored in the energy buffer and it always is below the storage capacity, $B_{i}\leq B$. Each sensor has limited energy buffer with equal storage capacity, which should be enough to provide at least one packet delivery or acceptance. The energy storage capacity is determined by physical material and storage technology, which is not the main point of our consideration in the work. Harvesting energy will be stored in an energy buffer at first, if a node is in the ideal state, until a packet arrives or if the buffer is full.

In the work, $P_{i}\left(t\right)$ indicates the ability of a harvesting energy device extracting energy from the environment. A high $P_{i}\left(t\right)$ value means more energy was extracted from the ambient conditions. For a sensor with solar panel as an energy supplement, the $P_{i}\left(t\right)$ is affected by three factors: lighting conditions, the size of panel, and the energy conversion efficiency. The energy conversion efficiency is influenced by energy conversion technology, for instance, at present, the solar-energy conversion efficiency is about 10%–30% [16]. We hypothesize that the power density (per square centimeter), the size of panel (square centimeter), and energy conversion efficiency are $\lambda_{t}$, $s_{panel}$, *γ*, respectively. Therefore, the $P_{i}\left(t\right)$ can be concluded as:

(6) $$p_{i}\left(t\right)=\lambda_{t}\times s_{panel}\times \gamma$$

For a solar panel as the energy source, the power density varies with weather conditions. Obviously, more power can be provided for a sensor on a sunny day and the power density can reach nearly 250,000 lux. Table 1 shows the power density in different weather conditions.

In R-WSNs, a sensor is expected to achieve energy-neutral operation, its energy used is always less than the energy harvested. The following equation should be satisfied for energy consumption.

(7) $$w_{i}\leq E_{i}$$

### 3.4. Data Aggregation Model

To incorporate data aggregation into the geometric routing model, we adopt the foreign-coding model [65] scheme. Specifically, we assume a node *i* is able to compress the data originating at its upstream neighbor *j* using its local data. The compression ratio depends on the data correlation between node *i* and *j*, which is denoted by the correlation coefficient ρ(i,j)=1-H(i|j)/H(i), where H(i) is the entropy coded data rate of the information at node *i*, and H(i|j) is the conditional entropy coded data rate of the same information H(i) at node *i* given the side information H(j). Examples of correlation models include the Gaussian random field model [66] which assumes that the correlation coefficient ρ(i,j) decreases exponentially with the distance between nodes, or $\rho \left(i,j\right)=\frac{1}{1+d(i,j)}$, and the inverse model [65] which assumes the data correlation is inversely proportional to the Euclidean distance between nodes, or $\rho \left(i,j\right)=exp(-\alpha \times d^{2}\left(i,j\right))$, where, *α* is data correlation parameters. Higher *α* means smaller data correlation, and vice versa.

### 3.5. Data Transmission Model

Let $S_{i}=\left\{j|d_{i,j}\leq R,j\in N\right\}$, where *R* is the radius of the node *i*’ transmission range. According to the geometric routing, only those neighbors that are closer to the sink node s can serve as the downstream nodes. Let us denote this set of downstream neighbors as $\vec{S_{i}}=\left\{j|d_{j,s}<d_{i,s},j\in S_{i}\right\}$. Similarly, the set of upstream neighbors is denoted as $\overset{\leftarrow }{S_{i}}=\left\{j|d_{j,s}\geq d_{i,s},j\in S_{i}\right\}$. Note that in case a node has no neighbors that are closer to the sink node than itself, we encounter a problem known as “local maximum” where the node fails to find routing path to the sink node according to geometric routing. A few solutions have been proposed for this problem [67,68]. However, the consideration of these solutions is beyond the scope of this paper. In the following, we assume that the downstream neighbor set $\vec{S_{i}}$ non-empty for all i∈N.

**Figure 4:**
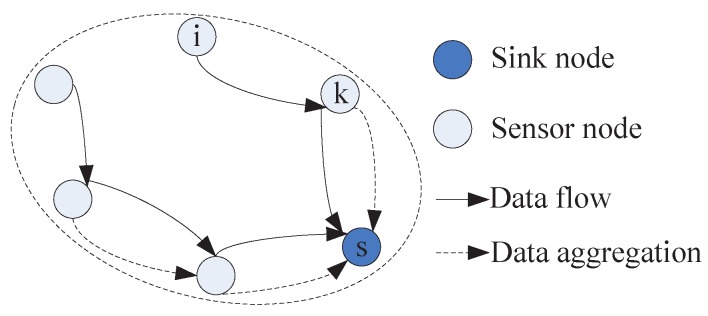
The process of data aggregation.

To further illustrate the process of data aggregation, an example is provided, which is shown in Figure 4. The data aggregation and routing work as follows. A sensor node *j* is ready to send data to its downstream neighbor node *s*. The data from node *j* contains two parts, one parts is aggregation data or transit data passed from node *i* (which has been encoded by node *i* or its upstream nodes), no further encoding is performed, which is directly forwarded to the downstream neighbors. Another part is the raw data of node *j*, it is encoded with the local information. All this transit traffic is forwarded to the downstream neighbors.

### 3.6. Topology Control Model

The topology control model is utilized for achieving two purposes in this work. First, the data transmission latency from source node to destination will be reduced or minimized by adjusting the transmission range of sensors flexibly. There is one more point, we should touch on: the energy utilization efficiency will be improved or maximized by neighbors’ selection strategies, rather than by saving power of the sensor node. Considering the limited energy storage ability, a sensor cannot always be able to conserve energy. Therefore, since the energy can be replenished continually, a sensor will consume the energy as soon as possible only if its energy can support its operation.

**Figure 5:**
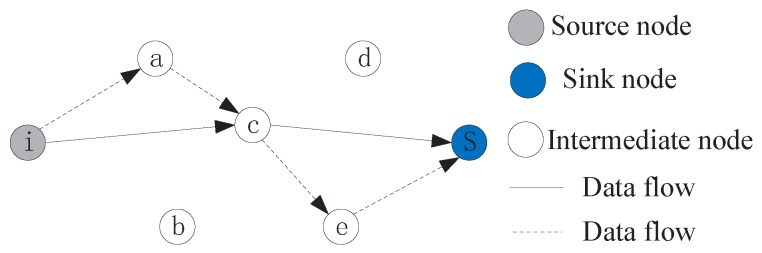
Topology control for data transmission.

To further illustrate the operation of the topology control model, an example is provided, which is shown in Figure 5. The sensor *i* senses the surroundings and generates packets, which will be forwarded to the destination sink *S* through multiple-hop data transmission. In traditional WSNs, by the Equation (2), we know that a sensor always select a closer neighbor as its next-hop node for data receiving in order to save energy. Therefore, data packets will be transmitted to sink *S* along the routing path {i,a,c,e,S}. While, in R-WSNs, considering the energy of sensor *i* can be replenished continually, it may select sensor *c* as its net-hop node only if it has enough energy to support this operation. The new routing path {i,c,S} can be established for data transmission. A packet is forwarded to sink *S* through two hops in the new path, while it is four hops in the original path. Therefore, the End to End delay for data transmission is reduced dramatically depending on the topology control model, which is significantly different to traditional WSNs.

## 4. Design and Method

### 4.1. Description of the Data Aggregation Problem

**Figure 6:**
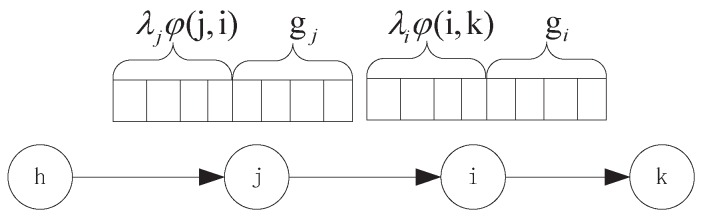
The aggregated transit traffic from a sensor to another sensor.

In R-WSNs, in the process of data transmission from packets generated by source nodes to these packets accepted by sink, packets will be encoded and aggregated in each sensor with local packets for reducing the data traffic. We let $\lambda_{i}$ denote the data aggregation rate of node *i* and φ(j,i) denotes the aggregated transit traffic rate at node *i* from its upstream node *j*. The aggregated transit traffic of node *i* is a superposition of two parts: the transit traffic passed from the upstream nodes, and the raw data originated from the upstream nodes that is to be encoded using the local information, as shown in Figure 6. That is,

(8) $$\lambda_{i}=\sum_{i,j\in V,j\in \vec{S_{i}}}\left(\lambda_{j}\varphi \left(j,i\right)+g_{j}\left(1-\rho \left(j,i\right)\right)\right)$$

where, $g_{j}$ and ρ(j,i) denote the data generation rate of node *j* and the data correlation between node *i* and *j*, respectively. For sensor *j*, the data traffic $\sum_{i,j\in V,j\in \overset{\leftarrow }{S_{i}}}\lambda_{j}\varphi \left(j,i\right)+g_{j}$ will be transmitted to its downstream sensor *i*, while these packets will be aggregated by sensor *i* firstly and send new data traffic $\sum_{i,j\in V,j\in \vec{S_{i}}}\left(\lambda_{j}\varphi \left(j,i\right)+g_{j}\left(1-\rho \left(j,i\right)\right)\right)$ to next-hop sensor along the path subsequently. The next-hop sensor that receives the packet will compress the packets by the Equation (8), so forth, until it reaches the destination.

In this work, multiple sinks are deployed for balancing the power of the networks, which is recognized and used wildly in WSNs. Generally, these sinks obtain the same function and status in the work. The data from a source node will be transmitted to any sink finally by one or multiple-hops. For each node, the outflow equals or is less than the inflow and generation data from it due to some redundant information removed from data traffic. In the process of packets transmission, a data leak is not considered. Therefore, the data flow traffic for node *i* can be expressed as:

(9) $$\sum_{i,j\in V,j\in \overset{\leftarrow }{S_{i}}}\left(\lambda_{j}\varphi \left(j,i\right)+g_{j}\left(1-\rho \left(j,i\right)\right)\right)=\sum_{i,j\in V,j\in \vec{S_{i}}}\lambda_{i}\varphi \left(i,j\right)$$

where, ρ(j,i)∈[0,1], when a sensor only forward packets for its upstream neighbor to next-hop sensor without any packet generation, data fusion does not occur and ρ(j,i)=0, while the two sensors have the exact same information and ρ(j,i)=1, which indicates that the packets are duplicated from that of another sensor. Let $w_{i}$ denote the fraction of power consumption for node *i* in each time unit. We have

(10) $$\begin{array}{cc} w_{i}= & e_{g}\times g_{i}+e_{idle}+e_{l}+e_{r}\sum_{j\in S_{i}}\left(\lambda_{j}\varphi \left(j,i\right)+g_{j}\right)+e_{t}\sum_{j\in S_{i}}\left(\lambda_{i}\varphi \left(i,j\right)+g_{i}\right) \end{array}$$

### 4.2. Description of the mAximum Data Collection Rate

In R-WSNs, the data collection rate of a sensor is affected directly by the amount of remaining energy extracted from ambient conditions. More energy supplement means that more packets will be collected. According to the Equations (7) and (10), for a sensor *i*, the maximum data collection rate per unit of time is formulated as:

(11) $$\begin{array}{cc} g(i)= & \frac{E_{i}-\left(e_{idle}+e_{r}\sum_{j\in \overset{\leftarrow }{S_{i}}}\left(\lambda_{j}\varphi \left(j,i\right)+g_{j}\right)\right)}{e_{g}}-\frac{e_{l}+e_{t}\sum_{j\in \vec{S_{i}}}\left(\lambda_{i}\varphi \left(i,j\right)+g_{i}\right)}{e_{g}} \end{array}$$

where, $e_{l}$ is used for a sensor to listen channel before data delivery with a fixed value for each time transmission. Hence, it is affected significantly by data flow, i.e., a sensor does not need to broadcast its existence when the channel is idle if there are no packets to deliver. Now, we let $e_{l}=k\times \sum_{i,j\in V,j\in S_{i}}f_{i,j}$. The energy depletion for listening and transmission can be expressed as:

(12) $$e_{l}+e_{t}\left(i,j\right)=\left(k+e_{t}\right)\times \sum_{i,j\in V,j\in S_{i}}f_{i,j}$$

Since *k* is fixed and $e_{t}$ is variable, we can let $e_{tr}$ represents the $k+e_{t}$. In fact, the energy utilization in idle state is a fixed value and small with less than 1 mW in per unit of time, which is only about 1/100 that of transmission and neglected in our work. Hence, the Equation (11) can be rewritten as:

(13) $$\begin{array}{cc} g(i)= & \frac{E_{i}-e_{r}\sum_{j\in \overset{\leftarrow }{S_{i}}}\left(\lambda_{j}\varphi \left(j,i\right)+g_{j}\right)}{e_{g}}-\frac{e_{tr}\sum_{j\in \vec{S_{i}}}\left(\lambda_{i}\varphi \left(i,j\right)+g_{i}\right)}{e_{g}} \end{array}$$

**Figure 7:**
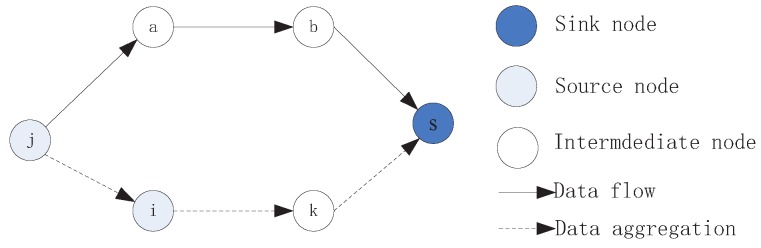
Different data transmission model.

From the Equation (13), we can observe the data collection rate for a sensor is affected significantly by the amount of its received data. The available energy is mainly consumed for at least two parts: receiving and transmitting. When more packets are accepted by a sensor, its data generation rate declines due to limited power supplement, which is an objective and obvious phenomenon in WSNs and R-WSNs. However, completely distinct strategies are adapted in them and a sample is provided for elaborating different routing schemes, as shown in Figure 7.

We assume node *j* and node *i* are source nodes and generate packets by sensing the environment periodically. There are at least two transmission schemes for delivering packets to the sink *s*. One of them is that packets can be forwarded along the link path {j,i,k,s}, where sensor *i* aggregates the packets from an upstream neighbor *j* with its self generated data and forwards it to the next-hop adjacent sensor until reaching the sink *s*. This scheme is widely and recognized and used in traditional WSNs, which can reduce the amount of packets by removing redundant information and reducing energy consumption, effectively contributing to extended network lifetime.

Another transmission scheme is that data from sensor *j* and *i* are delivered along paths {j,a,b,s} and {i,k,s}, respectively. Both raw data generated by node *j* and *i* will be transmitted to destination without considering data aggregation. The main differences to the two schemes can be summarized separately. For the first strategy, the advantage is energy consumption reduced with fewer packets by aggregating data. However, on the one hand, the data generation rate of node *i* declines. On the other hand, nodes *a*, *b* do not participate in the data transmission procedure, although their power is replenished repeatedly. For the second strategy, even though total energy consumption increases, the data collection rate of sensor *i* and sensors usage efficiency for node *a*, *b* are improved. Therefore, for improving data collection rate, the latter data delivery method is more suitable to R-WSNs with better performance compared to the first method.

The data collection rates vary over time as ambient condition is changed and are different to each other, and are significantly affected by energy harvesting rate and energy depletion rate. It is crucial to improve the data flow traffic for a sensor with minimum data collection rate. Therefore, we firstly find the minimum data collection rate of sensors. Secondly, we use the topology control and data fusion schemes. Lastly, we chose appropriate routing protocol to improve the data generation rate. Precisely, the objective is to find an optimal maximum minimum data collection rate, which will be defined in the following section.

### 4.3. Distribute Algorithm for Maximizing Data Collection Rate

The goal of the maximum data collection rate routing protocol is to deliver all the data packets generated by sensor nodes to base stations as soon as possible, subject to node harvesting energy, node capacity and link capacity constraints such that the data flow until the first (set of) sensor node with minimum data generation rate is maximized followed by data flow until the second (set of) sensor node with minimum value is maximized, and so on. Now, we formulate this problem as a line programming, which is given by:

(14) $$\begin{array}{c} Max\;g_{i}\\ Subject\;to:\\ \sum_{j\in \overset{\leftarrow }{S_{i}}}\left(\lambda_{j}\varphi \left(j,i\right)+g_{j}\left(1-\rho \left(j,i\right)\right)\right)=\sum_{j\in \vec{S_{i}}}\lambda_{i}\varphi \left(i,j\right) \end{array}$$

(15) $$\begin{array}{c} e_{r}\sum_{j\in \overset{\leftarrow }{S_{i}}}\left(\lambda_{j}\varphi \left(j,i\right)+g_{j}\right)+e_{tr}\sum_{j\in \vec{S_{i}}}\left(\lambda_{i}\varphi \left(i,j\right)+g_{i}\right)\\ +e_{g}g_{i}\leq E_{i} \end{array}$$

(16) $$\begin{array}{c} \lambda_{i}=\sum_{i,j\in V,j\in \vec{S_{i}}}\left(\lambda_{j}\varphi \left(j,i\right)+g_{j}\left(1-\rho \left(j,i\right)\right)\right) \end{array}$$

(17) $$\begin{array}{c} \sum_{j\in \overset{\leftarrow }{S_{i}}}\varphi \left(j,i\right),\sum_{j\in \vec{S_{i}}}\varphi \left(i,j\right)\leq R_{i},\forall i,j\in V \end{array}$$

(18) $$\begin{array}{c} \varphi \left(j,i\right),\varphi \left(i,j\right)\geq 0,\forall i,j\in V,j\in S_{i} \end{array}$$

(19) $$\begin{array}{c} 0\leq g_{i}\leq R_{i} \end{array}$$

(20) $$\begin{array}{c} E_{i}\leq B,E_{1}=W_{1} \end{array}$$

(21) $$\begin{array}{c} \lambda_{1}=g_{1} \end{array}$$

The first set of constraints in Equation (14) ensures that the inflow after aggregating with raw generation data equals the outflow. The second constraint in Equation (15) ensures that nodes do not consume more energy than they collect, which includes the energy consumption in sensing, packet transmissions, and packet receptions. The third constraint in Equation (16) formulates the $\lambda_{i}$ of node *i*. Equations (17)–(18) state that the data flow and available energy do not go below zero and do not go above the link capacity or battery capacity. The set of constraints in Equation (19) ensures that the data collection rate of a node is positive and finite. Equation (20) states that the available energy in the system initialization is the initial battery level. The last Equation (21) states the data aggregation rate equals data collection rate for a leaf or edge node without upstream neighbors.

According to the Equation (12), the first set of constraints models the data flow conservation at each node. We change the variable to q=1/g, which indicates the generation time for each unit of the packet. We define decay (q(i)) as the inverse of data collection rate (q(i)=1/g(i),g(i)>0). Therefore, maximum data collection rate can be converted to the minimum time per unit of packet generation. We obtain an equivalent linear programming formulation.

Here again, the flow conservation and power conservation constraints should be satisfied first. There are additional constraints that enforce all $q_{i}$ to be equal. Again we consider a quadratic objective function that is strictly convex in the $q_{i}$. Also, to ensure that the dual function is differentiable, we restrict the domain to $0\leq q_{i}\leq q$, for some loose upper bound *q*. In addition, we use a simple approach similar to that used in [69]. We change the primal objective function to $q_{i}^{2}$, since minimizing $q_{i}$ is the same as minimizing $q_{i}^{2}$. This is the optimization problem such that the maximum problem to maximize data collection rate is converted to the minimum problem to minimize data generation time for per unit of byte information, which will be solved in a distributed manner. We can interpret the above problem as minimizing the maximum ratio of time elapsed to collet a packet at a node. The linear programming problem for Equation (22) is NP-hard and considerably difficult to solve directly. Hence, a dual model will be utilized for replacing the original problem.

(22) $$\begin{array}{c} Min\;q_{i}^{2}\\ Subject\;to:\\ \sum_{j\in S_{i}}\left(\lambda_{i}\varphi \left(i,j\right)-\lambda_{j}\varphi \left(j,i\right)\right)=\frac{1-\rho (j,i)}{q_{j}}\\ h\leq q_{i}\left(E_{i}-\sum_{j\in S_{i}}\left(e_{r}\lambda_{j}\varphi \left(j,i\right)+e_{tr}\lambda_{i}\varphi \left(i,j\right)\right)\right)\\ q_{j}\left(\lambda_{i}-\sum_{j\in \vec{S_{i}}}\lambda_{j}\varphi \left(j,i\right)\right)=1-\rho \left(j,i\right)\\ h=e_{r}+e_{tr}+e_{g}\\ q_{i}=q_{j}\\ \sum_{j\in \overset{\leftarrow }{S_{i}}}\varphi \left(j,i\right),\sum_{j\in \vec{S_{i}}}\varphi \left(i,j\right)\leq R_{i},\forall i,j\in V\\ \varphi \left(j,i\right),\varphi \left(i,j\right)\geq 0,\forall i,j\in V,j\in S_{i}\\ 0\leq q_{i}\\ E_{i}\leq B,E_{1}=W_{1}\\ \lambda_{1}=1/q_{1} \end{array}$$

### 4.4. Lagrangian Dual Problem

We construct the dual problem by introducing Lagrange multipliers $\nu_{i}$ for the flow conservation constraint and $\mu_{i}$ for the data collection rate constraint at each node *i*. This results in the Lagrangian.

(23) $$\begin{array}{c} L\left(Q,f,\mu ,\nu \right)=q_{i}^{2}\\ +\sum_{i\in V}\nu_{i}\left\{\sum_{j\in S_{i}}\left(\lambda_{i}\varphi \left(i,j\right)-\lambda_{j}\varphi \left(j,i\right)\right)-\frac{1-\rho (j,i)}{q_{j}}\right\}\\ +\sum_{i\in V}\mu_{i}\left\{q_{i}-q_{j}\right\}\\ =q_{i}^{2}+\sum_{i\in V}\left(q_{i}\left(\mu_{i}-\mu_{j}\right)-\sum_{j\in S_{i}}\nu_{i}\frac{1-\rho (j,i)}{q_{i}}\right)\\ +\sum_{i\in V}\sum_{j\in S_{i}}\nu_{i}\left(\lambda_{i}\varphi \left(i,j\right)-\lambda_{j}\varphi \left(j,i\right)\right) \end{array}$$

The dual function is given by

(24) $$\begin{array}{c} g(\lambda ,\nu )\\ =\underset{0\leq q_{i}\leq q}{\inf}\{L\left(Q,f,\mu ,\nu \right)|h\leq q_{i}(E_{i}-\sum_{j\in S_{i}}(e_{r}\lambda_{j}\varphi \left(j,i\right)\\ +e_{tr}\lambda_{i}\varphi \left(i,j\right)))\}\\ =\underset{0\leq q_{i}\leq q,\lambda_{i}}{\inf}\{q_{i}^{2}+\sum_{i\in V}\left(q_{i}\left(\mu_{i}-\mu_{j}\right)-\sum_{j\in S_{i}}\nu_{i}\frac{1-\rho (j,i)}{q_{i}}\right)\\ +\sum_{i\in V}\sum_{j\in S_{i}}\nu_{i}\left(\lambda_{i}\varphi \left(i,j\right)-\lambda_{j}\varphi \left(j,i\right)\right)|h\leq q_{i}(E_{i}\\ -\sum_{j\in S_{i}}\left(e_{r}\lambda_{j}\varphi \left(j,i\right)+e_{tr}\lambda_{i}\varphi \left(i,j\right)\right))\} \end{array}$$

### 4.5. Subgradient Algorithm

We will use the subgradient algorithm to solve the dual problem. During the $k^{\prime }$th iteration of the subgradient algorithm, each node solves the following convex quadratic program with variables $q_{i},\lambda_{i}$, for $i\in V,j\in S_{i}$.

$$\begin{array}{c} Min\;q_{i}^{2}+\sum_{i\in V}\left(q_{i}\left(\mu_{i}-\mu_{j}\right)-\sum_{j\in S_{i}}\nu_{i}\frac{1-\rho (j,i)}{q_{i}}\right)\\ +\sum_{i\in V}\sum_{j\in S_{i}}\nu_{i}\left(\lambda_{i}\varphi \left(i,j\right)-\lambda_{j}\varphi \left(j,i\right)\right)\\ Subject\;to:\\ h\leq q_{i}\left(E_{i}-\sum_{j\in S_{i}}\left(e_{r}\lambda_{j}\varphi \left(j,i\right)+e_{tr}\lambda_{i}\varphi \left(i,j\right)\right)\right) \end{array}$$

The subgradient of -d at $\left(\mu^{k},\nu^{k}\right)$ is given by

(25) $$\begin{array}{c} \gamma_{\mu_{i}}^{\left(k\right)}=q_{j}^{\left(k\right)}-q_{i}^{\left(k\right)}\\ \delta_{\nu_{i}}^{\left(k\right)}=1-\rho \left(j,i\right)-\sum_{j\in S_{i}}\left(\lambda_{i}^{\left(k\right)}\varphi \left(i,j\right)-\lambda_{j}^{\left(k\right)}\varphi \left(j,i\right)\right) \end{array}$$

Hypothesis: the $\mu^{\left(0\right)},\nu^{\left(0\right)}$ are initial values of Lagrange multiplier, after *k* times of iteration.

(26) $$\begin{array}{c} \mu_{i}^{\left(k+1\right)}={\left(\mu_{i}^{\left(k\right)}-\theta_{k}\gamma_{\mu_{i}}^{\left(k\right)}\right)}_{+},\forall i=1,2,\ldots ,m\\ \nu_{i}^{\left(k+1\right)}={\left(\nu_{i}^{\left(k\right)}-\theta_{k}\delta_{\nu_{i}}^{\left(k\right)}\right)}_{+},\forall i=1,2,\ldots ,p \end{array}$$

where, $\theta_{k}$ denotes the iteration step. For any θ>0, it exist a value *r*, when $\theta_{k}\in \left(0,r\right)$.

(27) $$\begin{array}{c} \theta_{k}>0,\theta_{k}\to 0,\sum_{k=1}^{\infty }\theta_{k}=\infty \end{array}$$

The algorithm converges to the maximum value d(μ,ν). If it is a strong duality, $d^{\left(k\right)}=d^{*}\left(\mu^{\left(k\right)},\nu^{\left(k\right)}\right)$ converges to an optimal solution for the original problem. The subgradient algorithms are used to solve it in a distributed manner. The algorithm is utilized for calculating the upper bound of data flow under ideal conditions rather than establishing a detailed routing path. In actual application, the network throughput will be lower than the result value of our method.

## 5. Results and Discussion

Simulation of our algorithm for R-WSNs was done by Matlab software, with up to 200–400 nodes and 3–8 sinks being randomly deployed in a 500 m × 500 m to 1000 m × 1000 m square field. The maximum communication range of each node is set to be 80 m. All nodes’ energy devices are rechargeable with 20 cm${}^{2}$ square size solar panel, and transmission powers are adjustable. The solar panel will be affected by cloudy or sunny environments, as well as the angle of sunlight, as solar radiation may change at any time, along with time or climate. Every two nodes can communicate with each other directly within their transmission range. Energy leakage and the case of signal loss of sensor are not considered in our work. $\theta_{k}\approx 1/3500$, $\rho \left(i,j\right)=exp(-\alpha \times d^{2}\left(i,j\right)),\alpha \in \left[0.001,0.01\right]$, low value of *α* indicates high correlation, vice versa. Every data point in simulation figures is obtained by averaging 50 runs with different random seeds, node deployment and node working schedules, and some related simulation parameters are provided in Table 2. In the simulation, part of the information of the sensor stems from an actual environment, such as, the signal intensity, signal interference, etc.

**Figure 8:**
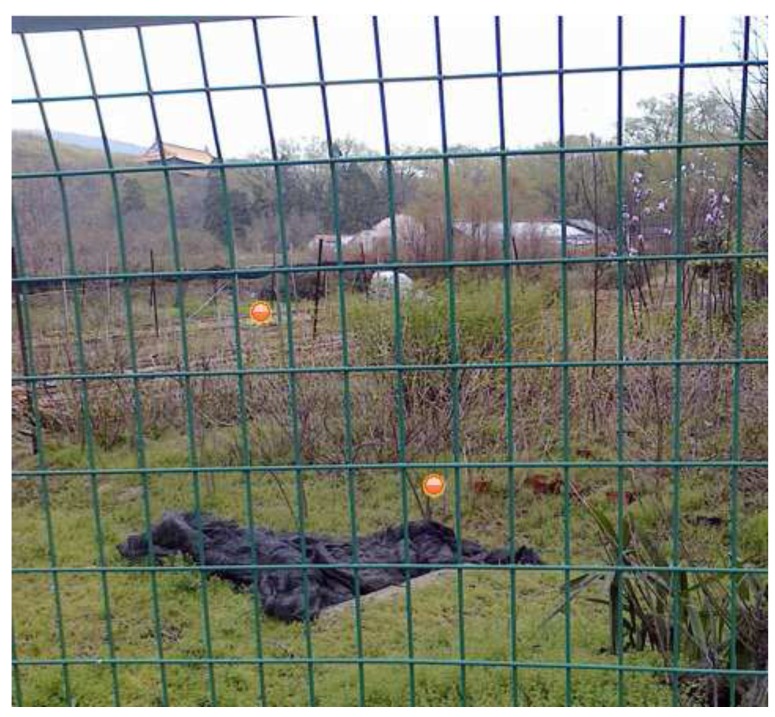
The outdoor scenes for two sensors.

**Figure 9:**
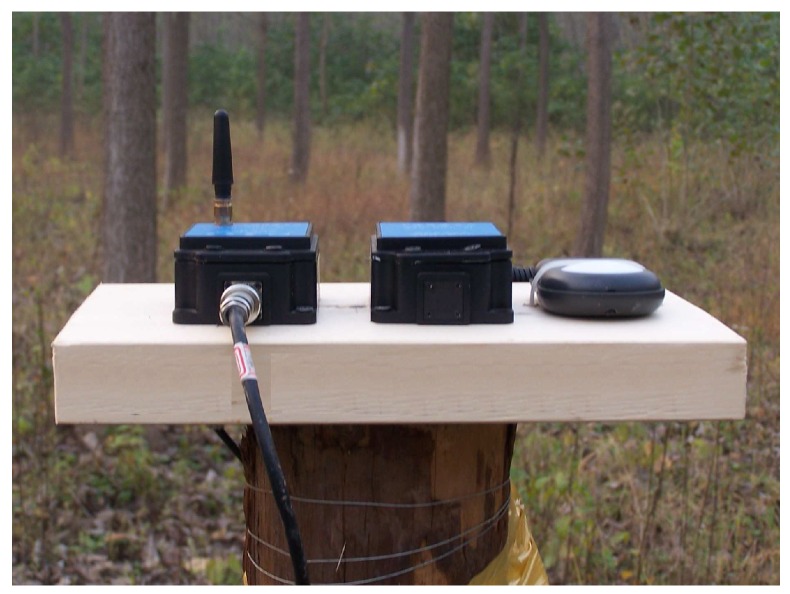
A sensor node is deployed in outside.

What we use consists of a solar panel optimized for outdoor use, two eZ430-RF2500T target boards and one AAA battery pack, which is rechargeable and can be recharged repeatedly. The target board comprises the TIMSP430 microcontroller, CC2500 radio transceiver and an on-board antenna. The CC2500 radio transceiver operates in the 2.4 GHz band with data rate of 250 kbps and is designed for low power wireless applications. The harvested energy is stored in EnerChip, a thin-film rechargeable energy storage device with low self-discharge manufactured by Cymbet, as is shown in Figure 8 and Figure 9.

### 5.1. System Implement

Due to available energy constrained, it is a common practice to deploy multiple sinks for collecting packets from all sources in realistic application, which means there are at least two advantages compared with just one sink. Firstly, in the multiple sinks scenario, since packets generated by sensors are only needed to be forwarded to any one sink, the degree of routing path from source node to sink will be shorted significantly. Secondly, a limited number of sensors are distributed around the sink and it often represents a bottleneck for data transmission because all packets from sources have to be forwarded to these sensors before reaching a destination, while it will relieve this pressure for data forwarding with more than one sink. These two strong points will save energy and improve data generation rate.

**Figure 10:**
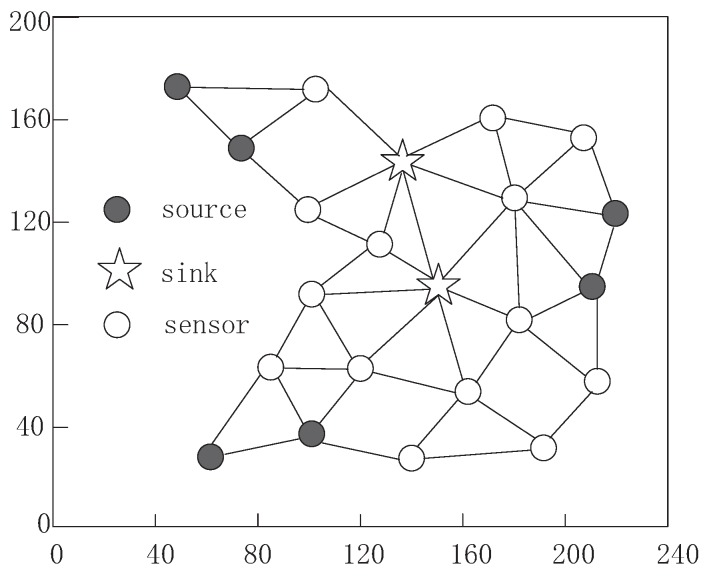
The routing process using maximum data collection rate scheme.

**Figure 11:**
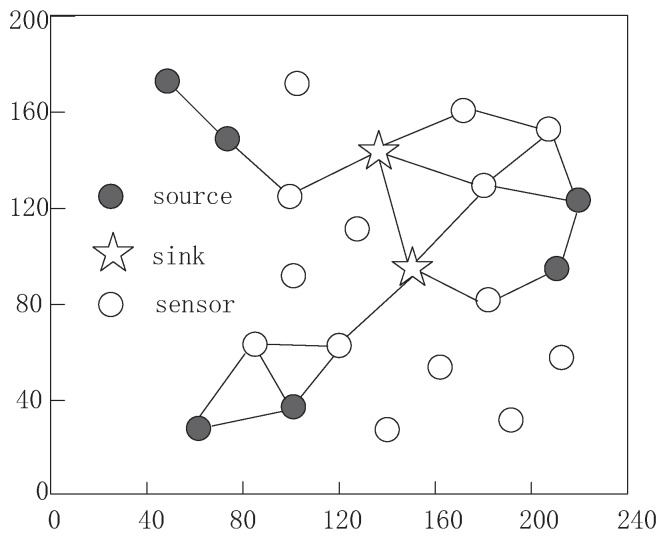
The routing process using minimum energy consumption scheme.

Figure 10 and Figure 11 show the routing paths established using different communication models when 20 sensors and 2 sinks deployed in experiment scenario with a 200 m × 240 m square area, which contains 6 source nodes, a sensing signal and other sensors undertaking forwarding tasks. Figure 10 presents the routing paths for all sources to reach at least one sink based on maximum data collection rate of our algorithm. By considering the limited energy supplement, a source node still chooses a closer base station as its destination; however, the packets from sources may travel through all intermediate nodes and the energy consumption will be balanced to all nodes in order to improve sensor usage efficiency.

From the Figure 11, we can observe that only some of the nodes participate in the routing path formation. The Figure 11 shows that the routing process is based on the minimum energy consumption [70]. A source node will create a route path to the nearest base station with smallest link distance between each of the two adjacent nodes in order to reduce energy consumption, which is recognized and widely used in traditional WSNs. When a source sends its data to another source, data aggregation occurs and total packets are reduced, which is attributed to low power expenditure. There are at least four advantages of this scheme, such as, high data delivery speed, better system stability, low data transmission delay and low energy expenditure. However, it is designed for traditional WSNs based on fixed battery with constrained energy, which must be correct in R-WSNs.

### 5.2. System Performance Comparison

In order to further understand of the performance of our algorithm for Maximizing data collection rate (Mdcr) under network settings, in this section, we provide two schemes for performance comparison, including an optimal distributed lexicographic rate assignment (Dlex) designed for R-WSNs [53] and an Energy-efficient data aggregation tree (Edat) applied in WSNs. In [53], the authors propose distributed algorithms for determining the routing structure and amount of flows on each link without considering data fusion. It involves updated rate computation using optimal lexicographic rate assignment. In this method, nodes first compute their maximum rate using an initial rate procedure. Subsequently, they send a control packet containing the flow id and the maximum achievable rate to their next hop nodes.

In [71], Edat is constructed for energy efficiency, in which energy management is the main task and data aggregation is a secondary aim for energy efficiency in routing design. There is limited literature on maximum data generation rate routing based on data aggregation in R-WSNs. At the same time, the two methods were widely recognized and used for maximum data collection rate in R-WSNs and data fusion in WSNs, which can be attributed to the decision to select them for performance comparison in our work.

**Figure 12:**
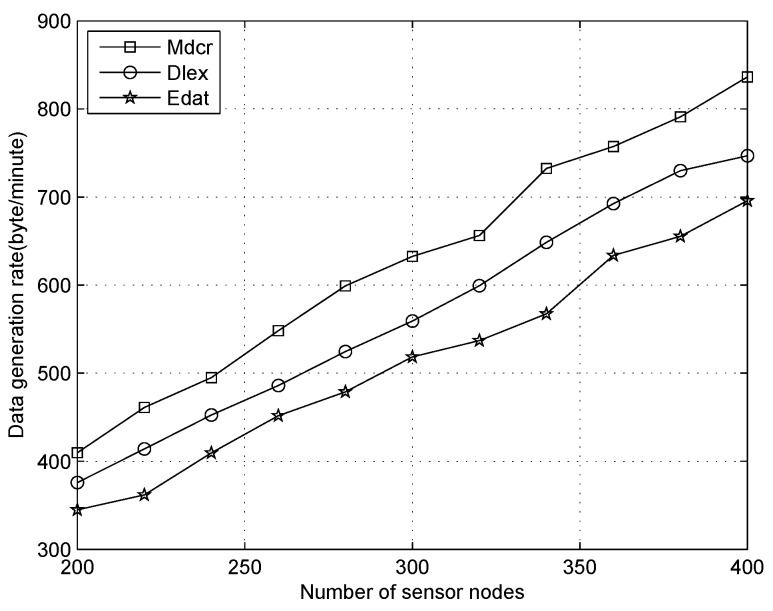
Data generation rate with number of nodes increasing when duty cycle is 1%.

**Figure 13:**
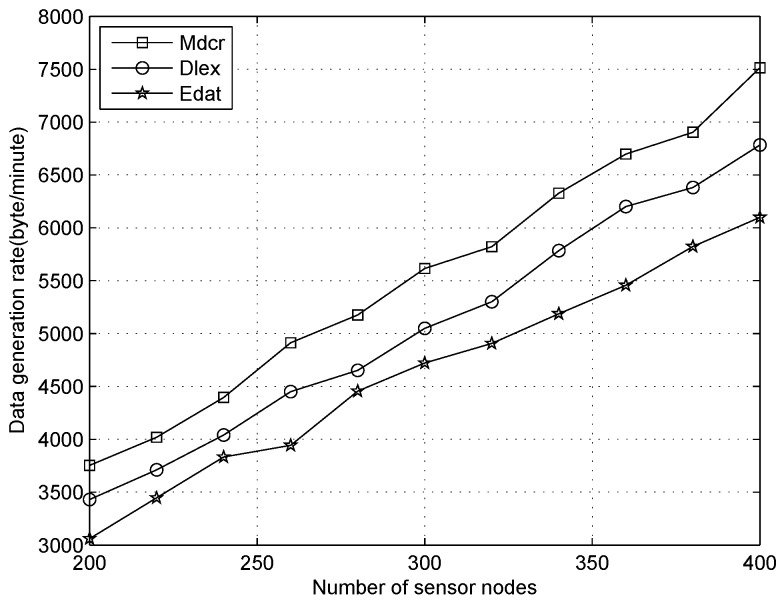
Data generation rate with number of nodes increasing when duty cycle is 10%.

**Figure 14:**
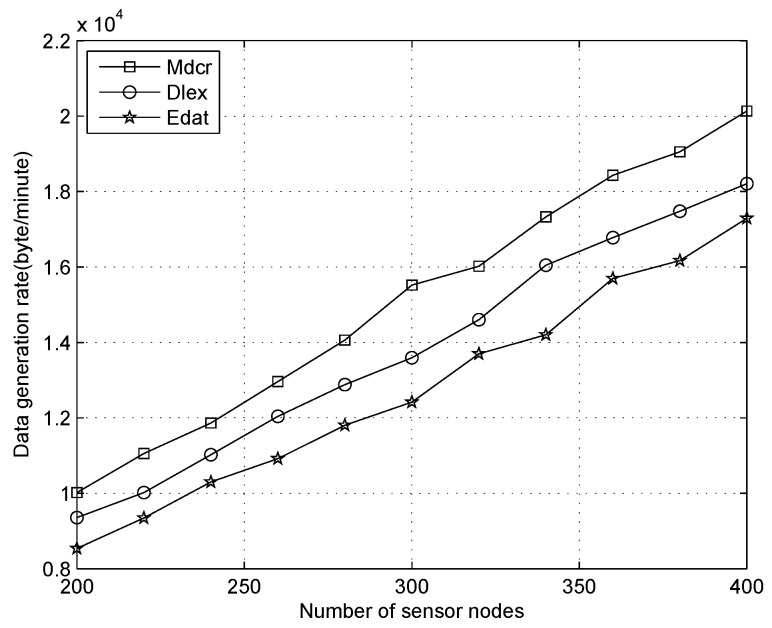
Data generation rate with number of nodes increasing when duty cycle is 30%.

We first compare the data generation rate between our algorithm and other two schemes under the distinct number of sensors and only one sink, where the average node’s duty-cycles are 1%, 10%, 30%, respectively, as shown in Figure 12, Figure 13 and Figure 14. From Figure 12, we can observe the data collection rates increase for all algorithms and that of our algorithm is slightly higher than that of other algorithms with the sensor density being improved for all different node’s duty-cycles. Our algorithm is utilized for calculating the upper bound of data flow under ideal conditions rather than establishing a detailed routing path. In actual application, the network throughput will be lower than the result value of our method. From Figure 12, we can see that the data generation rate of our algorithm is about 9% and 15% higher than that of the Dlex and Edat scheme, respectively.

Since high sensor density indicates that more packets will be sensed and forwarded to the destination, the data collection rate will be improved, and power expenditure will be balanced between all sensors if we append more sensor nodes to the monitoring field in a reasonable number of sensors. Despite this, sensors usually keep in a low duty cycle and collision is scarce: if too many sensors are deployed in a smaller area, the collision will be critical and retransmission is more frequent, which causes serious energy waste. In this work, we only consider an ideal condition that a reasonable number of sensors were deployed outdoors and collision was ignored.

Comparing Figure 12 with Figure 14, when the duty cycles exceed 30 times in Figure 14 compared with that in Figure 12, we can see that the data collection rate is improved significantly and it is about 25 times. Higher data collection rate is obtained with improved duty cycles, which indicates that a sensor will be in an active state more times and data transmission occurs more frequently. The deeper reason for higher duty cycle is that more energy is extracted from the environment. Therefore, a sensor will generate more packets utilizing extra energy, from which we observe that more data will be collected on a sunny day than in cloudy weather.

**Figure 15:**
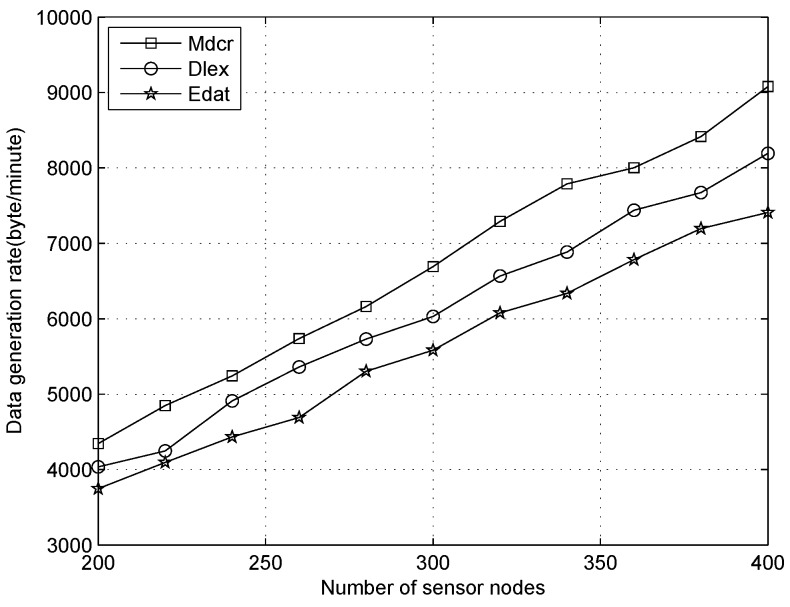
Data generation rate with number of nodes increasing when data correlation α=0.001.

**Figure 16:**
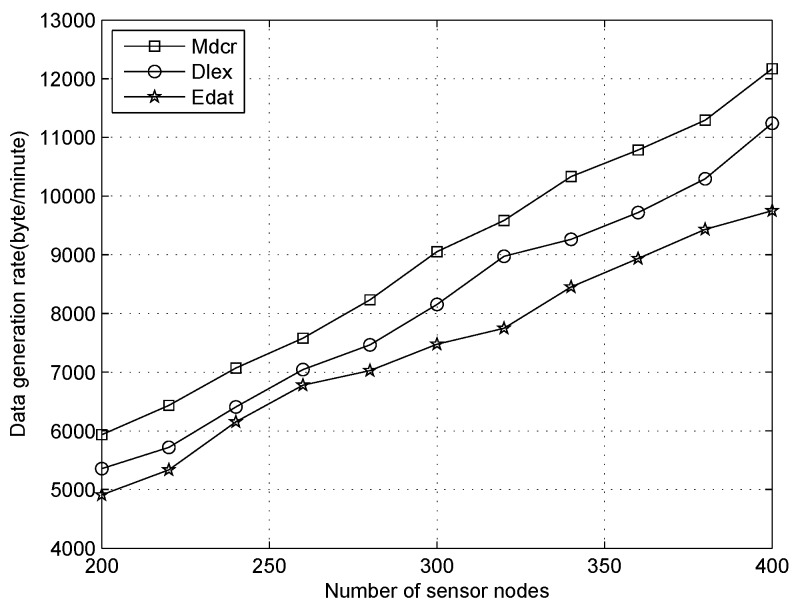
Data generation rate with number of nodes increasing when data correlation α=0.005.

**Figure 17:**
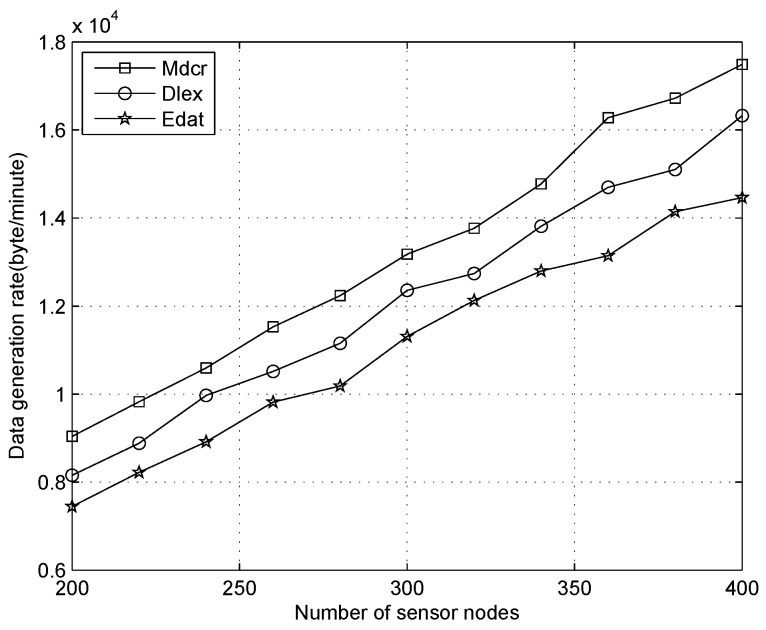
Data generation rate with number of nodes increasing when data correlation α=0.01.

We next analyze the data collection rate for all sensors under three data correlation settings (α=0.001, 0.005 and 0.01), as shown in Figure 15, Figure 16 and Figure 17. From Figure 15, we can observe that the data flow rate in three algorithms increases with the number of nodes increasing in three different scenarios, with our algorithm performing a little better than the Dlex and Edat. As the number of nodes increases in the area, it affects the node density and delivery ratio. The overall raw data rate is proportional to the number of nodes in the network. More packets were generated in the network and the data aggregation rate improved greatly. At the same time, the nodes’ density increased, which also drives the network topology from sparse to dense and the data correlation between neighboring nodes becomes higher, so more redundant information can be removed through data aggregation.

With improving *α*, the data correlation and the data redundancy decrease, which indicates that the data variance increases, such as, packets come from two sensors with long distance or different scenario. Hence, more packets will be transmitted to the sink and data aggregation rate is also improved. When α=0.001, the data correlation coefficient and data redundancy are relatively high, packets can be compressed with high ratio and data collection rate increases due to a decrease in the power being used for data forwarding. While α=0.01, the data correlation coefficient is low, which indicates that the packets are very different to each other and data is hard to compress. Therefore, original raw data will be forwarded to the sink and there will be more power expenditure for this procedure, which causes low data generation rate.

To further illustrate the performance of data collection based on the topology controlling for our scheme we introduce a new algorithm that uses a cooperative repeater to improve data collection in low power generation for solar-powered wireless sensor networks [72]. In [72], the authors propose a repeater role to some nodes for improving the data collection performance, which only relays data from the other nodes without transmitting its own data. The reason for selecting the methods for performance comparison is that high data collection rate is the purpose and the scene is also subject to solar-powered WSNs. At the same time, the selected method is recognized and widely used for improving data collection rate in WSNs.

**Figure 18:**
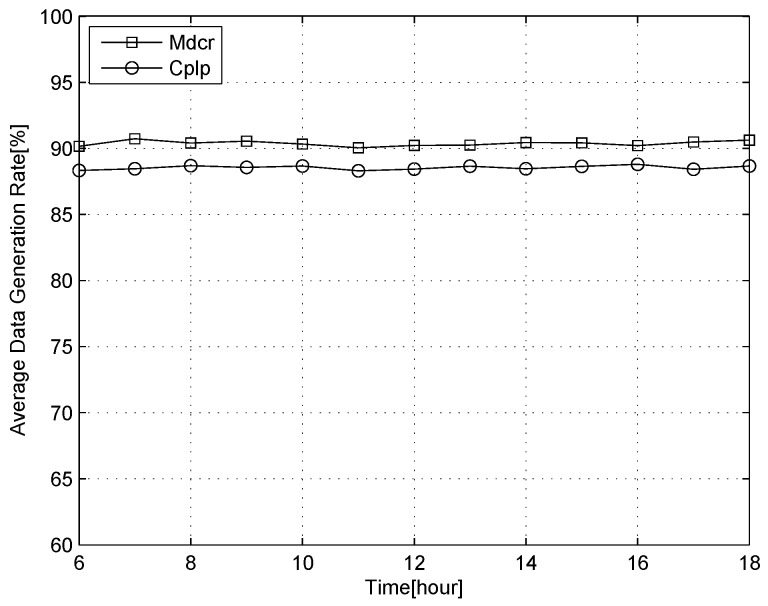
Comparison of the average data collection rate for a day when the probability of sunny day.

Figure 18 shows the comparison of the average data collection rate for a day when the probability of a sunny day is high in our scheme and in [72], which can be abbreviated as Cplp (Cooperative repeater for low power WSNs). We can see that the average data collection rate of the scheme sustains a high lever compared to Cply on a sunny day. This is because the topology controlling techniques are utilized for adjusting its communication paths continually, while this method has been considered in Cplp.

**Figure 19:**
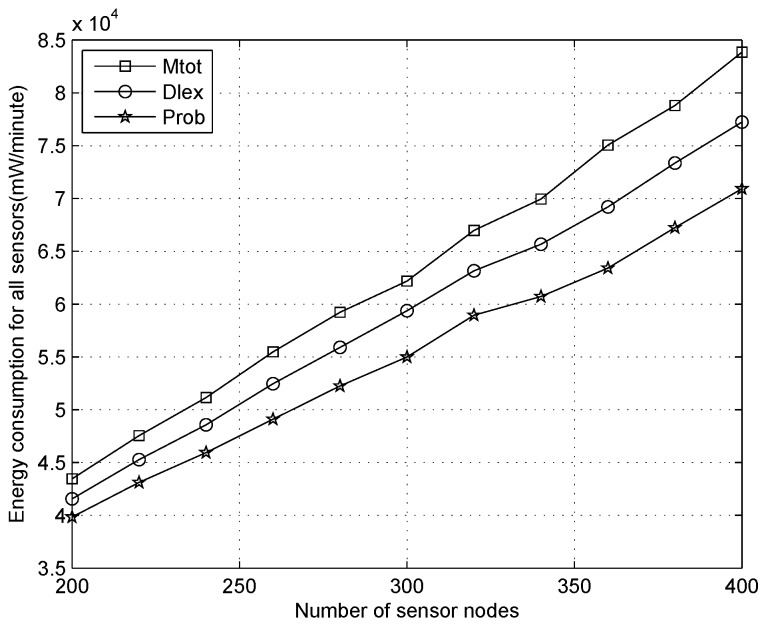
Energy consumption with the number of sensor nodes increasing when the duty cycle is 1%.

**Figure 20:**
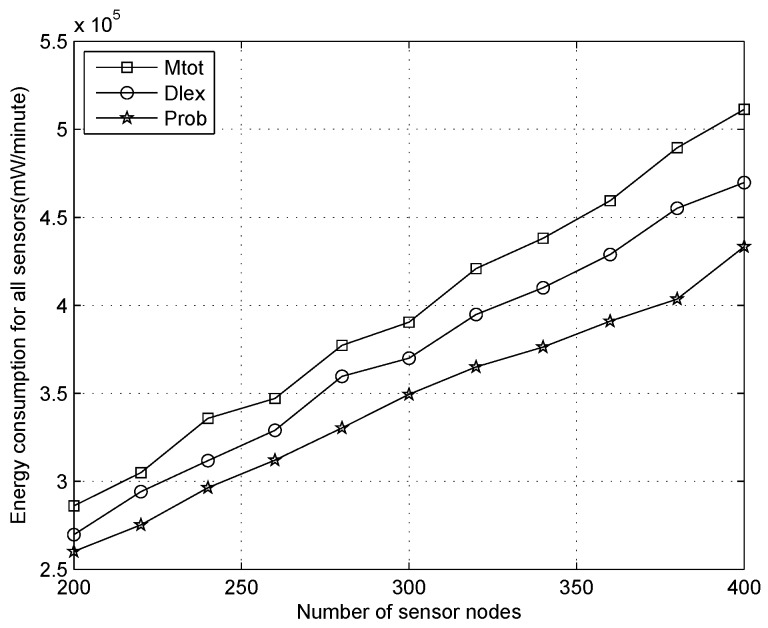
Energy consumption with the number of sensor nodes increasing when the duty cycle is 10%.

**Figure 21:**
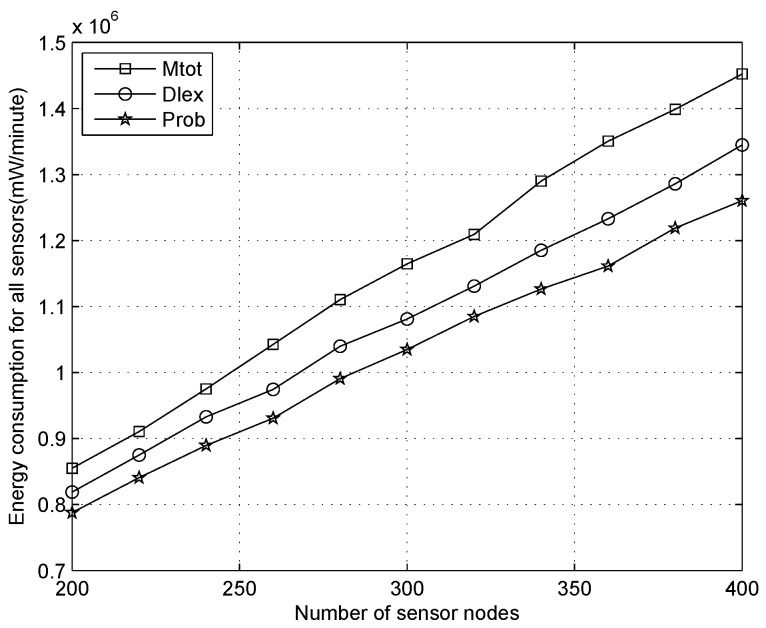
Energy consumption with the number of sensor nodes increasing when the duty cycle is 30%.

We analyze the total energy depletion for all sensors with the number of nodes increasing when the duty cycle is 1%, 10%, 30%, as shown in Figure 19, Figure 20 and Figure 21, respectively. From Figure 19, we can observe that the energy depletion increases gradually when more sensors are added to the scenario, and our algorithm will consume more energy than other methods for any number of sensors. This does not mean that energy is wasted and there is a network lifetime reduction, which is the point of traditional WSNs. On the contrary, it demonstrates an outstanding performance for our algorithm because the power of each node can be replenished continually, which is significantly different to traditional WSNs, where saving energy is considered prior to routing design, e.g., the Edat scheme obtains better performance with respect to saving energy.

Comparing Figure 19 with Figure 21, when the duty cycles are raised 30 times in Figure 21 compared to Figure 19, we can see that the total energy expenditure increases significantly, i.e., about 20 times. When the duty cycles improved due to data collection rate increases, the total energy consumption will improved, which indicates that more energy is extracted from the environment. From another point of view, when more energy is supplied for a sensor, it will be more lively in the active state and undertake more tasks, such as, increased packet generation. Therefore, on a sunny day, we will improve the duty cycles of sensors with more energy and consume the energy as soon as possible.

**Figure 22:**
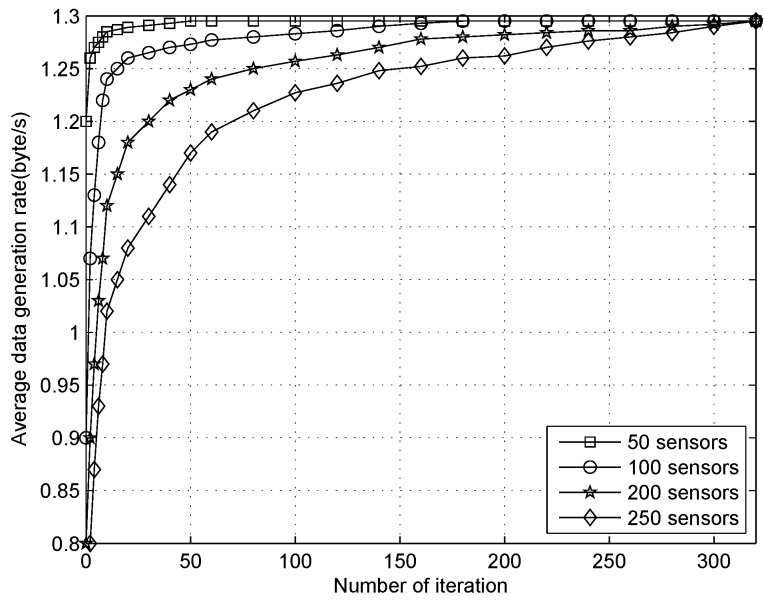
The iterations for data generation rate converges to maximum value.

Figure 22 shows that the data generation rate for each sensor converges to maximum value in our algorithm when the number of sensors are 50, 100, 200, respectively. For fewer nodes, the algorithm converges faster to the maximum generation rate. As the number of nodes increase, convergence of iterations needs to increase gradually. This is because, with the expansion of a network scale, the total sample space is growing, and algorithms often need to go through more iterations to achieve convergence. As it can be seen from Figure 22 , when the number of nodes is 50, 100, 200 and 250, the number of times to reach 95% of maximum generation rate value needs: 55, 120, 220, 300 iterations to be performed, repectively. Overall, it will converge to maximum value with iterations no more than double the size scale of the networks in all scenarios, which is affordable in terms of the computing ability of the sensors.

**Figure 23:**
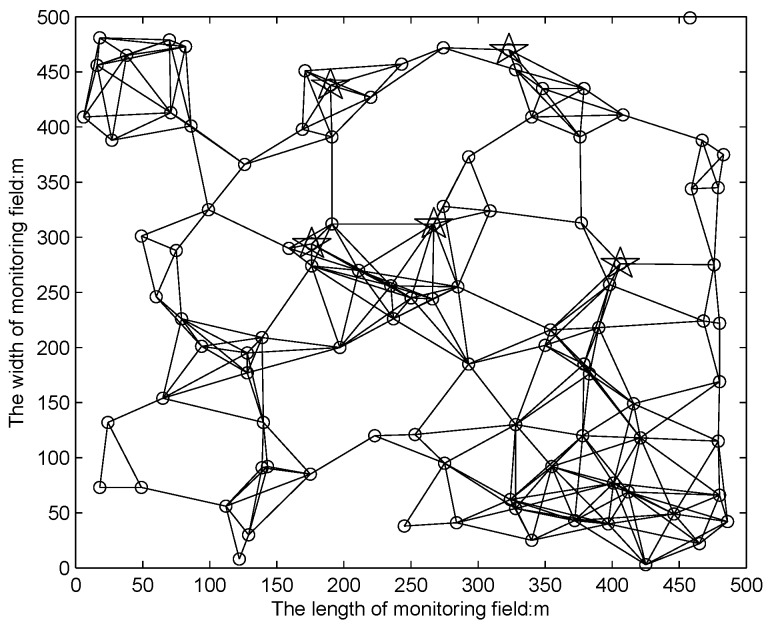
Connectivity graph with 100 nodes and 5 sinks deployed in a 500 m × 500 m.

The connectivity graph is provided in Matlab for expressing the routing paths for all sensors with connection to any one sink, where 100 sensors and 5 sinks are deployed in a 500 m × 500 m square field, which is shown in Figure 23. By considering the limited energy supplement, a source node still chooses a closer base station as its destination. However, the packets from all sources may travel through all intermediate nodes in the network area before they reach a sink, and the communication energy consumption will be balanced to all nodes in order to improve sensor utilization efficiency.

## 6. Conclusions

In this work, we first define the network system, energy expenditure and replenished model, data aggregation scheme, and elaborate on the packet delivery process. The unique characteristics of R-WSNs pose a big challenge for packet’s transmission. On the one hand, data collected by many sensors is based on common phenomena, and hence there is a high probability that this data has some redundancy. On the other hand, due to energy being replenished continually and limited energy storage capacity, it might not always be beneficial to conserve energy when a network can harvest excessive energy from the environment. Therefore, reasonable energy usage can improve data collection rate and sensor usage efficiency.

In order to improve data generation rate, we propose an algorithm to compute an upper data generation rate based on data fusion that maximizes it as an optimization problem for a network. First and foremost, we formulate it as a linear programming problem subject to the flow and energy conservation constraints. On top of that, a dual problem by introducing Lagrange multipliers is constructed. Last but not least, a subgradient algorithm is used to solve it in a distributed manner. Through extensive simulation and experiments, we demonstrate that our algorithm is efficient to maximize data collection rate in rechargeable wireless sensor networks.

## Figures and Tables

**Table 1:** The power density in different weather condition.

Weather Condition (at 12:00 noon)	The Power Density
cloudy day inside	4000–7000 lux
cloudy day outside	8000–12,000 lux
sunny day inside	30,000–50,000 lux
sunny day outside	>100,000 lux

**Table 2:** The simulation parameters.

Parameter	Value
*area*	500 m × 500 m to 1000 m × 1000 m
*packet*	512 byte
*sinks number*	3–8
*sensors number*	200–400
$\varepsilon_{elec}$	50 nJ
$\varepsilon_{amp}$	100 pJ/bit/m${}^{2}$
*the size of panel $s_{panel}$*	20 cm
*energy conversion efficiency γ*	30%
*transmission range R*	80 m
Batteryrange(Bmin;Bmax)	0, 10 MJ
